# Research on Pig Sound Recognition Based on Deep Neural Network and Hidden Markov Models

**DOI:** 10.3390/s24041269

**Published:** 2024-02-16

**Authors:** Weihao Pan, Hualong Li, Xiaobo Zhou, Jun Jiao, Cheng Zhu, Qiang Zhang

**Affiliations:** 1 School of Information and Artificial Intelligence, Anhui Agricultural University, Hefei 230036, China; panweihao17114212@stu.ahau.edu.cn (W.P.); zxb@ahau.edu.cn (X.Z.); jiaojun2000@ahau.edu.cn (J.J.); 2 Institute of Intelligent Machines, Chinese Academy of Sciences, Hefei 230031, China; hlli@iim.ac.cn; 3 Department of Biosystems Engineering, University of Manitoba, Winnipeg, MB R3T 5V6, Canada; qiang.zhang@umanitoba.ca

**Keywords:** pig, filter, GMM-HMM, DNN-HMM, sound recognition

## Abstract

In order to solve the problem of low recognition accuracy of traditional pig sound recognition methods, deep neural network (DNN) and Hidden Markov Model (HMM) theory were used as the basis of pig sound signal recognition in this study. In this study, the sounds made by 10 landrace pigs during eating, estrus, howling, humming and panting were collected and preprocessed by Kalman filtering and an improved endpoint detection algorithm based on empirical mode decomposition-Teiger energy operator (EMD-TEO) cepstral distance. The extracted 39-dimensional mel-frequency cepstral coefficients (MFCCs) were then used as a dataset for network learning and recognition to build a DNN- and HMM-based sound recognition model for pig states. The results show that in the pig sound dataset, the recognition accuracy of DNN-HMM reaches 83%, which is 22% and 17% higher than that of the baseline models HMM and GMM-HMM, and possesses a better recognition effect. In a sub-dataset of the publicly available dataset AudioSet, DNN-HMM achieves a recognition accuracy of 79%, which is 8% and 4% higher than the classical models SVM and ResNet18, respectively, with better robustness.

## 1. Introduction

The sounds pigs make have a variety of meanings, which can reflect their behavioral characteristics. It is very important to predict pig states by identifying the information in pig sound signals. In the early days, pig sounds were mainly recognized artificially by technicians, which was not only costly but also inefficient. With the rapid development of modern information technology, artificial intelligence, signal processing and other technologies, the combination of sound feature analysis and computer technology can improve the efficiency and contribute to the development of the pig industry [1].

Some progress has been made in studying pig sounds so far. In the aspect of pig sound preprocessing, Yin et al. [2] used two fusion strategies, feature fusion and classifier fusion, in preprocessing to resist the complex sound environment of pigsties, so as to improve the classification accuracy and achieve the purpose of accurately recognizing the coughing sounds of pigs. In the study of Wu et al. [3], improved empirical mode decomposition-Teager energy operator (EMD-TEO) cepstral distance was used for the endpoint detection of pig sound signals, and the accuracy could reach 90.293%. In the research of pig sound recognition, Wu et al. [4] proposed a pig sound classification method based on the dual roles of signal spectrum and speech, which achieved a classification accuracy of 93.39% in the pig sound classification task. Ji et al. [5] extracted audio features including root-mean-square energy (RMS), mel-frequency cepstral coefficients (MFCCs), and zero-crossing rate (ZCR) from the audio signals of pigs, extracted visual features including localized binary pattern (LBP) and histogram of gradients (HOG) using constant Q transform (CQT) spectrograms, and fused the audio with the visual features to detect whether the pigs were coughing or not, with an accuracy of 96.45%. Sun et al. [6] extracted the Meier frequency cepstrum coefficients of hogs as audio feature parameters and utilized a BP neural network recognition model to recognize the coughing sound of hogs with a recognition accuracy of 85.33%. In a study by Chung et al. [7], support vector machine was adopted to classify and identify pig sound signals in different diseases, which provides an effective reference for defining the disease status of farmed pigs. In the study by Liu et al. [8], Hidden Markov Model (HMM) was used to recognize pig cough sounds, with a recognition rate of 80%, indicating that the model can be used for the diagnosis of respiratory diseases in pigs. Cho et al. [9] transformed one-dimensional sound signals into two-dimensional gray images and brought them into a convolution neural network to detect wasting disease in pigs, with an accuracy of 96%. Shen et al. [10] used the fusion of acoustic and visual features to recognize the coughing sound of a pig by utilizing a recursive feature elimination algorithm based on random forests for acoustic feature selection and inputting the acoustic and visual features into a support vector machine. Liao et al. [11] proposed a classification model called TransformerCNN, which combines the advantages of CNN and Transformer to form powerful global feature perception and local feature extraction, and the model had an accuracy of up to 96.05% for domestic pig sound recognition. Yin et al. [12] proposed a classification algorithm based on fine-tuning the AlexNet model with spectrogram features, which utilizes the image recognition advantage of convolutional neural networks so as to identify the spectrogram of coughing sounds of pigs, and the overall recognition rate of the model is 95.4%. Shen et al. [13] proposed a new feature of Mel frequency cepstrum coefficients fused with a convolutional neural network (MFCC-CNN), which was obtained by fusing multiple frames of MFCCs with multiple single-layer CNNs to improve the recognition accuracy of coughing sounds of live pigs. Gong et al. [14] recognized pig cough sounds using an improved MFCC and vector quantization model, achieving a high recognition rate of 94.21%. Wang et al. [15] used an improved lightweight MobileNetV3_esnet model to recognize sows’ estrous and non-estrous sounds with 97.12% accuracy. Min et al. [16] extracted the time–frequency features of pig sounds, frequency domain resonance peak features and MFCC features and used a decision tree, the K-nearest neighbor algorithm and the support vector machine algorithm to classify them, and k-fold validation was carried out after classification, and the experiments showed that the classifiers had clear judgments of classifying the sound of coughing pigs and indicated that there was a need to further observe pig socialization behaviors through the sound features of pigs. The research on pig sound recognition all over the world mainly focuses on pig coughing, while pig sound recognition in other states has been rarely reported. In addition, in these papers, only a few types of pig sounds have been included, which are generally different from the target sound to be distinguished, and thus the learned recognition models cannot recognize the pig sounds in other states, which limits the practical use of these models.

**Figure 1:**
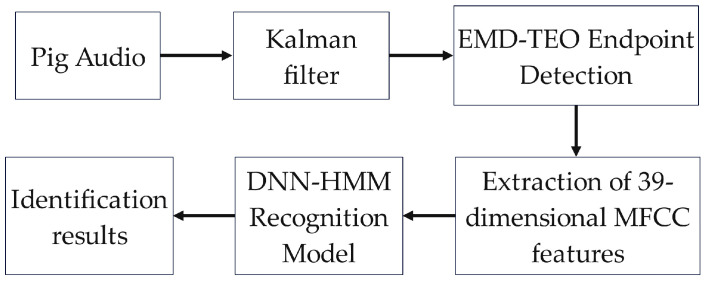
Overall flow chart of this study.

To compensate for the recognition of daily sound signals of healthy pigs, this study first employs Kalman filtering to denoise the collected pig sound signals. Subsequently, an improved EMD-TEO cepstral distance endpoint detection algorithm is applied to segment the pig sound, extracting the sound segments. The sound is then processed into 39-dimensional mel-frequency cepstral coefficients (MFCCs), which are used as a dataset for training within a DNN-HMM model, as shown in the flowchart in Figure 1. This approach harnesses deep neural networks to achieve more accurate recognition of sounds from five different healthy growth states in pigs.

## 2. Materials and Methods

### 2.1. Materials

In this study, the hardware system for acoustic signal acquisition and transmission was composed of the master controller NanoPc-T4, external iTalk-02 microphone, USB interface, etc. The computer hardware environment used in this study was equipped with an 11th Gen Intel(R) Core(TM) i5-1155G7 CPU running at 2.50 GHz. The operating system of the computer was Windows 10, and the software environment included Python version 3.8 and Matlab version 2016(a). The sound preprocessing section of this study was conducted using Matlab. The GMM-HMM training segment was performed using Python. In order to obtain a high-quality mono sound signal, all sound files were recorded in WAV format with PCM coding. The number of channels was set to 1, the sampling size to 16 bits, and the sampling rate to 44.1 kHz. The pig sounds used in the experiment were collected in Jing Huimeng Pig Farm in Mengcheng, Anhui Province, as follows. Ten adult finisher Landrace pigs were sequentially placed in a 2 m by 4 m quarantine pen. And the sound recorder was fixed on the pig pen, about 15 cm away from the right side of the pigs. Ten-hour pig sound signals were collected by the sound signal acquisition system in a relatively quiet space. When no pigs are present, the ambient noise level in the area is around 12 dB. Then, the sound samples undergo preprocessing steps such as Kalman filtering for noise reduction and endpoint detection, resulting in the acquisition of a substantial amount of pig vocalization segments. Those pig sound segments were divided into five categories, namely, eating sounds, humming sounds, estrous sounds, howling sounds and panting sounds, by consulting veterinarians and pig experts. Experts categorized the various sound segments manually based on their expert experience. A total of 2500 valid segments of pig sounds were used in the GMM-HMM training, including 480 eating sounds, 420 estrus sounds, 530 howling sounds, 570 humming sounds, and 500 panting sounds. The various types of sound were divided into training and testing sets in an 8:2 ratio.

### 2.2. Sound Signal Preprocessing and Feature Extraction

The collected sound signals were preprocessed before use to minimize the impact of noises in the environment to pig sound signals, obtain more stable sound signals and extract better sound features, thus generating a highly robust sound model.

#### 2.2.1. Kalman Filtering

As there are various noises in the pig farming environment [17], it is very important to select a reasonable filtering algorithm for the identification of pig sounds. In this study, the Kalman filtering algorithm was used to reduce the noises of the collected pig sounds [18,19], with the main sources of noise being pig pen exhaust fan noise, birds singing and machinery buzzing. It takes the linear minimum mean square error as the optimal estimation criterion and uses the state-space representation to establish the state equation of signals and noises. The state variables are continuously modified and predicted through the estimated value at the previous moment and the observed value at the current moment [20]. The filtering results of the dynamic system are obtained by iteration. The specific processes are as follows:

(1) Defining a system of discrete control processes $x_{k}$ that can be described by linear stochastic differential equations and the measured values of the system $z_{k}$:
  (1) $$x_{k}=Ax_{k-1}+Bu_{k}+W_{k}$$
  (2) $$z_{k}=Hx_{k}+v_{k}$$
  where $x_{k}$ is the system state at time *k*. $u_{k}$ is the control quantity of the system at time *k*. ***A*** and ***B*** are system parameters. $z_{k}$ is the measured value at time *k*. ***H*** is the parameter of the measurement system. $w_{k}$ is the process noise assumed to be Gaussian white noise and its covariance is *Q*. $v_{k}$ is the measurement noise assumed to be Gaussian white noise and its covariance is *R.*
(2) Using the process model of the system to predict the current state x(k|k−1) based on the previous state,
  (3) $$x(k|k-1)=Ax(k-1|k-1)+Bu_{k}$$
  where x(k−1|k−1) is the optimal result of the previous state.
(3) Updating covariance *P*,
  (4) $$P(k|k-1)=AP(k-1|k-1)A^{Τ}+Q$$
  where P(k|k−1) is the corresponding covariance of x(k|k−1). P(k−1|k−1) is the corresponding covariance of x(k−1|k−1). $A^{T}$ is the transpose matrix of A. Q is the covariance of the system process.
(4) The optimal estimated value x(k|k) of the current state k can be obtained by combining the predicted value and measured value:
  (5) $$x(k|k)=x(k|k-1)g_{k}(z_{k}-Hx(k|k-1))$$
  where $H^{T}$ is the transposition of H in its expression, and $g_{k}$ is the Kalman gain, defined as $g_{k}=P(k|k-1)H^{T}/(HP(k|k-1)H^{T}+R)$.
(5) In order to keep the Kalman filter running until the end of the system, the covariance x(k|k) in the state k should be updated:
  (6) $$P(k|k)=(I-g_{k}H)P(k|k-1)$$
  where I is a matrix of 1. P(k|k) will change to P(k−1|k−1) when the system enters the state *k* + 1.

#### 2.2.2. Endpoint Detection

Due to the silent section and noisy section in the collected pig sound signals, it is necessary to determine the starting point and end point of the sound signal so that the data quality and calculation efficiency can be improved [21].

**Figure 2:**
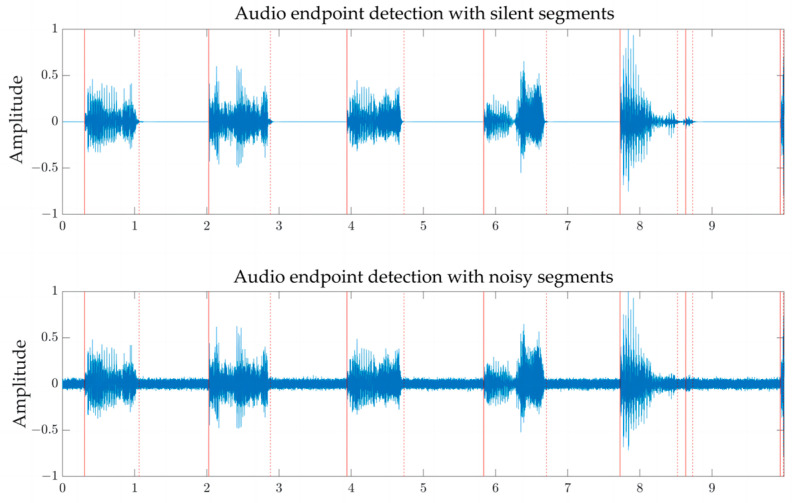
Endpoint detection results including silent segments or noisy segments.

As shown in Figure 2, the silent segments and noisy segments within a sound clip need to be removed. Therefore, an endpoint detection algorithm is required to identify the segments containing pig sounds. The solid red line in Figure 2 represents the starting point, while the dashed red line represents the endpoint.

This research draws on the improved cepstral distance endpoint detection algorithm of EMD-TEO designed by Wu Yawen et al. [3] to achieve this purpose:

(1) The EMD algorithm is used to decompose the denoised pig sound signal into multiple single-modal functions (IMFs).
(2) TEO is used to process the modal components with special meaning, obtain the energy spectrum of the modal components, and extract the characteristic frequency parameters of pig sounds.
(3) The short-time cepstral distance method is introduced to calculate the cepstral distance parameters to obtain more accurate endpoint values.
(4) The endpoint detection of two-level parameters is adopted to identify the start value and end value of the effective signal.

#### 2.2.3. Signal Feature Extraction

The original signals are time sequence signals of variable length, which have a high degree of redundancy in the time domain and cannot be directly used as the input of the learning algorithm. The sound feature extraction converts a sound signal into an indirect and logical feature vector, which is more discriminative and reliable than the actual signal [22].

**Figure 3:**
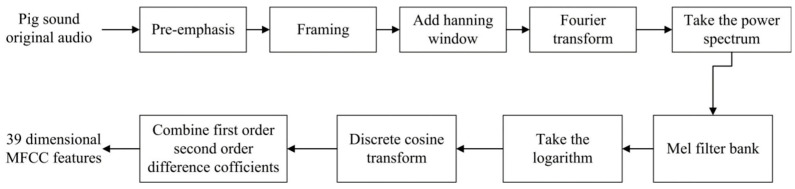
Flow chart of MFCC acoustic feature extraction.

The auditory system is similar to a filter bank [23], which has certain selectivity for sound waves of different frequencies. They are widely distributed in the low-frequency signal area but sparsely distributed in the high-frequency signal area. The mel frequency cepstral coefficient (MFCC) [24] is obtained by imitating the auditory mechanism. Mel filter energy can be obtained from the mel filter after pre-emphasis, framing, adding windows and fast Fourier transform (FFT) to the original pig sound signals, and its discrete cosine transform (DCT) is calculated to form a 13-dimensional MFCC reflecting the static characteristics of pig sounds. In the processes, the pre-emphasis coefficient is set to 0.98, and the frame length and frame overlap time are set to 20 ms and 10 ms, respectively. Then, the 39-dimensional MFCC is finally obtained by adding the first-order and second-order difference coefficients. The specific processes of MFCC extraction are shown in Figure 3.

### 2.3. Construction and Evaluation of Sound Classification Model

The sound characteristics of live pigs were used as the experimental materials to construct the sound recognition model for pigs.

#### 2.3.1. DNN-HMM Model

Hidden Markov Model (HMM) is a probabilistic graphical model [25], which describes two interdependent stochastic processes: the observable process and hidden Markov process. Through the calculation of probability, the maximum possibility is selected to estimate the output sequence of predicted sound. A deep neural network (DNN) is a traditional feed-forward artificial neural network with multi-layer hidden units [26]. In the DNN-HMM framework, HMM is used to describe the dynamic changes of sound signals while DNN is used to estimate the probability of observed features [27,28,29].

**Figure 4:**
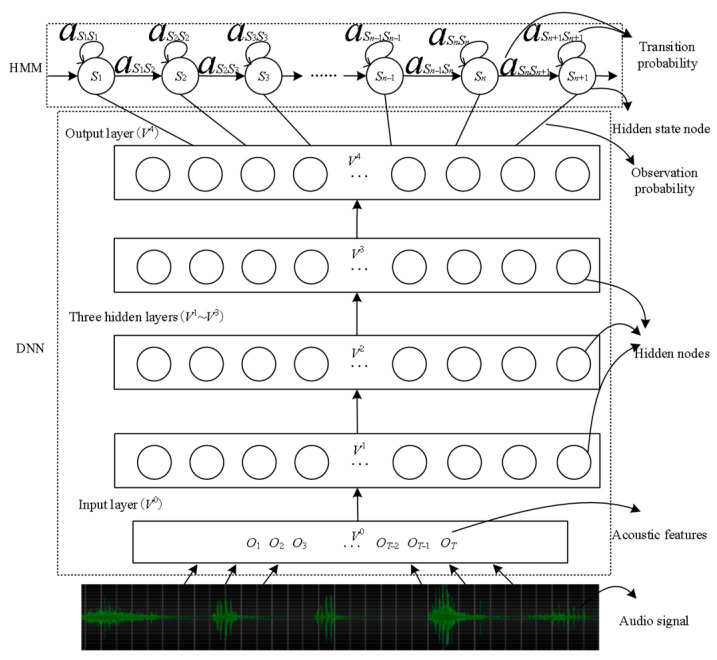
DNN-HMM model structure diagram.

Figure 4 shows a DNN-HMM structure with three hidden layers. The MFCC feature of the extracted speech signal is used as the input layer. Each of the three DNN hidden layers includes 128 hidden nodes. The output layer is connected to the HMM. The HMM includes several hidden states, and each hidden state can self-cycle or point to the next hidden node. It can be expressed by the posterior probability of the DNN output nodes corresponding to the states when it is necessary to calculate the observed value probability of a certain state of a phoneme on the acoustic characteristics of a frame. Since the decoding of HMM requires the likelihood probability, and the posterior probability from the output layer of DNN should be converted into the likelihood probability,

(7) $$p(x_{t}|q_{t})=p(q_{t}|x_{t})p(x_{t})/p(q_{t})$$

where $x_{t}$ is the observed value; $q_{t}$ is the state at time *t*; the resulting acoustic probability is expressed as follows:

(8) $$p(x|w)=\underset{q}{\sum }p(x,q|w)p(q|w)≅\max\pi (q_{0})\prod_{t=1}^{T}a_{qt-1qt}\prod_{t=0}^{T}p(q_{t}|x_{t})/p(q_{t})$$

where w is the possible recognition sequence obtained by the Viterbi algorithm; $p(q_{t}|x_{t})$ is calculated by DNN; $p(q_{t})$ is the state a priori probability; $\pi (q_{0})$ and $a_{q_{t-1}q_{t}}$ are the initial state probability and state transition probability determined by HMM.

#### 2.3.2. Training GMM-HMM

DNN-HMM consists of DNN, HMM and a priori probability distribution. Due to the phoneme-binding structure shared by DNN-HMM and GMM-HMM, a GMM-HMM system needs to be trained before training the DNN-HMM model [30]. DNN training tagging is generated by the GMM-HMM system and the Viterbi algorithm, and the quality of tagging will affect the performance of the DNN system, so the initial training model of GMM-HMM is very important.

The observed value probability of GMM-HMM is represented by GMM. GMM contains multiple Gaussian functions, namely the probability density function (PDF) [31]. Therefore, it is necessary to reevaluate the starting probability, transition probability, the weight of different PDFs in each state and the mean and variance of different PDFs in each state. Since the HMM with a left-to-right structure is adopted for sound recognition, the starting probability is set to [1, 0, 0, …, 0], which means that training can only start from the first state. Combined with the forward–backward algorithm of HMM, the following statistics are defined:

(9) $$\begin{array}{c} {\gamma_{t}}^{c}(j,k)=[\frac{\alpha_{t}(j)\beta_{t}(j)}{\sum_{j=1}^{N}\alpha_{t}(j)\beta_{t}(j)}][\frac{c_{jk}G({o_{t}}^{c},\mu_{jk},\sum_{jk})}{\sum_{k=1}^{K}c_{jk}G({o_{t}}^{c},\mu_{jk},\sum_{jk})}]\\ \;\;\;\;\;\;\;\;=\left\{\begin{array}{c} \frac{1}{p(o|\lambda )}\pi_{j}\beta_{1}(j)c_{jk}G({o_{1}}^{c},\mu_{jk},U_{jk}),t=1\\ \frac{1}{p(o|\lambda )}\sum_{i=1}^{N}\alpha_{t-1}(i)a_{ij}\beta_{t}(j)c_{jk}G({o_{1}}^{c},\mu_{jk},U_{jk}),t>1 \end{array}\right. \end{array}$$

where $\alpha_{t}(j)$ is the forward probability, the probability of seeing the observation sequence $\left\{o_{1},o_{2},\ldots ,o_{t}\right\}$ at time *t* and in hidden state $q_{t}$; $\beta_{t}(j)$ is the backward probability, the probability of generating the rest of the observation sequence $\left\{o_{1+t},o_{2+t},\ldots ,o_{T}\right\}$ from time *t +* 1 to the end of time *T* after being in *t* in hidden state $q_{t}$ at time *t*; $c_{jk}$ is the proportion assigned to the Gaussian component *k* for the observations associated with the state $s_{j}$; p(o|λ) is the marginal probability distribution; *G* is Gaussian function; $\gamma_{c}^{t}(j,k)$ is the weight. Based on the re-estimation formulas of HMM and GMM, the maximum likelihood (ML) [32] criterion and the Baum–Welch algorithm [33], the re-estimation formulas of expectation-maximization (EM) [34] of GMM-HMM parameters are generated as follows:

(10) $${a_{i}}_{j}=\frac{\sum_{c=1}^{C}\sum_{t=1}^{T_{c}-1}{\xi_{t}}^{c}(i,j)}{\sum_{c=1}^{C}\sum_{t=1}^{T_{c}-1}{\gamma_{t}}^{c}(i)}$$

(11) $$c_{jk}=\frac{\sum_{c=1}^{C}\sum_{t=1}^{T_{c}}{\gamma_{t}}^{c}(j,k)}{\sum_{k=1}^{K}\sum_{c=1}^{C}\sum_{t=1}^{T_{c}}{\gamma_{t}}^{c}(j,k)}$$

(12) $$\mu_{jk}=\frac{\sum_{c=1}^{C}\sum_{t=1}^{T_{c}}{\gamma_{t}}^{c}(j,k){o_{t}}^{c}}{\sum_{c=1}^{C}\sum_{t=1}^{T_{c}}{\gamma_{t}}^{c}(j,k)}$$

(13) $$\sum_{jk}=\frac{\sum_{c=1}^{C}\sum_{t=1}^{T_{c}}{\gamma_{t}}^{c}(j,k)({o_{t}}^{c}-\mu_{jk})({o_{t}}^{c}-\mu_{jk}{)}^{\prime }}{\sum_{c=1}^{C}\sum_{t=1}^{T_{c}}{\gamma_{t}}^{c}(j,k)}$$

where C is the number of training samples; $a_{ij}$ is the transition probability, which is not affected by GMM; $\xi_{t}^{c}(i,j)$ represents the probability *t* of being in hidden state $s_{i}$ at time *t* and being in hidden state $s_{j}$ at time t+1. Each of the characteristics of observation $o_{t}^{c}$ participates in the calculation of each Gaussian mean and variance, and its proportion is determined by $\gamma_{t}^{c}(j,k)$; $\mu_{jk}$ and $\sum_{jk}$ are estimates of the corresponding mean and variance.

In these processes, we need to set the appropriate number of iterations and threshold, so that a trained GMM-HMM model can be obtained when the model converges or reaches the maximum number of iterations.

#### 2.3.3. Supervised DNN Training

In the DNN-HMM model, DNN is used to simulate the posterior probability of hidden states of HMM under the given input observation states. We extract the HMM from GMM-HMM as the HMM part of DNN-HMM and replace GMM with DNN and take the argument label generated by the trained GMM-HMM model in the previous section as the input of DNN. We select the appropriate activation function and generate the output vector from Equation (14):

(14) $$v^{l}=f(z^{l})=f(W^{l}v^{l-1}+b^{l}),0<l<L$$

where $z^{l}=W^{l}v^{l-1}+b^{l}$ is the excitation vector. $v^{l-1}$, $W^{l}$ and $b^{l}$ are the activation vector, weight matrix and deviation coefficient matrix; *L* is the number of nerve layers; f(⋅) is the activation function for element-level calculation of the excitation vector.

Through the error back-propagation algorithm [35], the appropriate loss function is selected to update the weight and offset. The error of the output layer is transmitted from the hidden layer to the input layer in turn to achieve the transfer of the loss cost layer by layer, and the weight and offset parameters are adjusted in each layer, respectively, until the expected loss function value is almost not updated or reaches the minimum convergence state. The update of the weight $W^{l}$ and offset $b^{l}$ are determined by the following formulas [36]:

(15) $${W_{\left(t+1\right)}}^{l}={W_{\left(t\right)}}^{l}+\eta \frac{\partial J_{CE}}{\partial {W_{\left(t\right)}}^{l}}$$

(16) $${b_{\left(t+1\right)}}^{l}={b_{\left(t\right)}}^{l}+\eta \frac{\partial J_{CE}}{\partial {b_{\left(t\right)}}^{l}}$$

where η is the learning rate; ${W_{\left(t\right)}}^{l}$ and ${b_{\left(t\right)}}^{l}$ are the weight matrix and offset vector of the layer l updated at iteration t, respectively; ${W_{\left(t+1\right)}}^{l}$ and ${b_{\left(t+1\right)}}^{l}$ are the DNN model parameters obtained in t+1 training. $\frac{\partial J_{CE}}{\partial {b_{\left(t\right)}}^{l}}$ and $\frac{\partial J_{CE}}{\partial {W_{\left(t\right)}}^{l}}$ are the average weight matrix gradient and average offset vector gradient for layer l at iteration t.

#### 2.3.4. Construction and Test of Pig Sound Model

**Figure 5:**
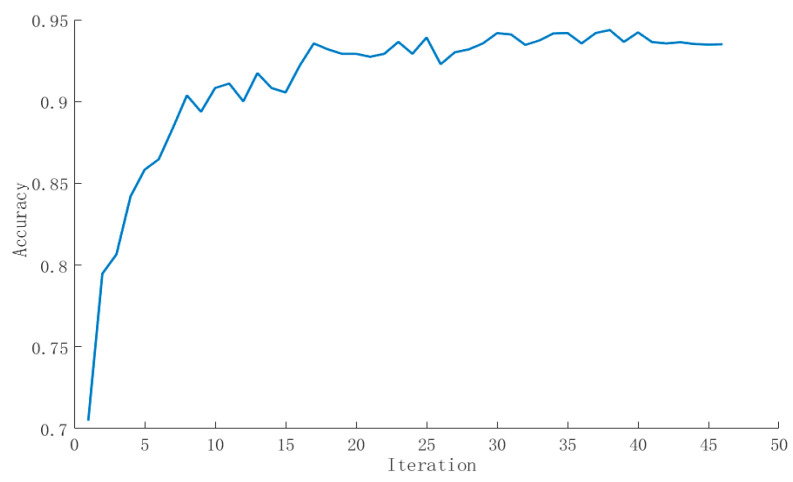
Variation curve of accuracy of live pig sound validation set.

**Figure 6:**
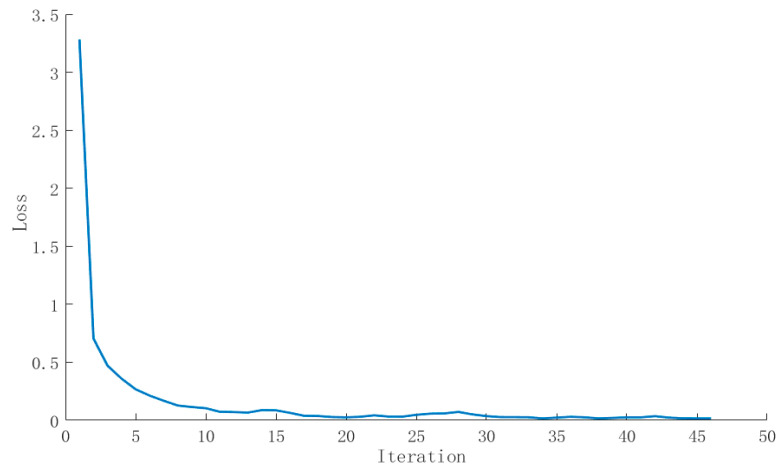
Variation curve of loss value of live pig sound validation set.

The 39-d MFCCs of pig sound samples were extracted as sound features and used to construct the DNN-HMM model. The specific processes are as follows:

(1) GMM-HMM training. Based on the theoretical knowledge in Section 2.3.2, five hidden states were used to simulate the pig sounds of eating, estrus, humming, howling and panting. The given initial probability length was the same as the number of states, the first element was 1, and the other elements were set to 0. The hidden state transition probability matrix was set to 5 × 5 and the total value to 1. The observation state transition probability matrix was initialized by evenly dividing the pig sound feature training samples and estimating their global mean and variance. The Baum–Welch algorithm was used to optimize and re-evaluate the GMM parameters, and the alignment information was obtained by the Viterbi algorithm to update the HMM parameters. In this process, the number of iterations was set to 40 and the convergence threshold is 10^−6^. After this process, based on the GMM-HMM system, the alignment between the data frame of the training sound and the corresponding states of the relevant syllable was achieved.
(2) Supervised DNN training. We imported the data labels aligned between the completed frames and the hidden states into DNN. In order to reduce network redundancy, splice was set to 3, which means that in addition to the current frame, three frames before and three frames after it were included to generate a 7-frame vector, so that the information of adjacent frames could be used to model the relationship between context features. Considering the sound number and feature dimension of training, a neural network with three hidden layers was set. In order to speed up the calculation, 128 nodes were set in each layer, and the softmax layer was set as the output layer. The output probability was obtained through forward propagation, and the error loss was calculated with the training label obtained in the first step. Throughout the process, the activation function used the rectified linear unit (ReLU) [37], whose expression is [38,39]

(17) f=max(0,z)

where z is the output of the fully connected network. The cross-entropy [40] (CE) loss function $J_{CE}$ adopted is defined as follows:

(18) $$J_{\mathrm{CE}}=-\sum_{i}\hat{y_{i}}\ln y_{i}$$

where i is the output layer node index; $\hat{y}$ is the real label generated by the GMM-HMM model and its activation function is $y_{i}$. The weight W and offset b are updated by the error back-propagation algorithm. We set the initial learning rate to 0.003 and the proportion of data in the verification set to 0.1. In order to speed up the training calculation, we set epoch to 10 and batch_size to 100, the maximum number of iterations to 200, and the convergence threshold to 10^−6^ and used the adaptive motion estimation (Adam) optimization algorithm to adjust the learning rate. In order to prevent overfitting, the early stopping method, which means that the training will stop when the performance of the model on the verification set begins to decline, was used. From the changes in accuracy (Figure 5) and loss value (Figure 6) of the pig sound verification set, it can be seen that the training loss of DNN continues to decline, and the accuracy of model verification also increases after multiple iterations. The final loss value is about 0.04, and the accuracy is about 93.5%.

(3) Model testing. After the DNN-HMM model was trained, we used the MFCC features extracted from the test sound samples corresponding to several pig behavior states to input into the five sound models $\lambda_{n}=(A,B,\pi ),(n=1,2,3,4,5)$ obtained from the training. The best hidden-state transition path of the test samples in the recognition model was searched through the Viterbi algorithm, and the cumulative output probability was calculated. Finally, we compared the output probability of the test sample in five models to obtain the sound recognition results.

#### 2.3.5. Performance Test Indices

The quality of the sound recognition system can be evaluated by the recognition rate. In this study, recall, accuracy and specificity were used as performance measures. The higher their values are, the better the model is. Their calculation formulas are as follows:

(19) $$recall=\frac{TP}{TP+FN}$$

(20) $$accuracy=\frac{TP+TN}{TP+FP+TN+FN}$$

(21) $$specificity=1-\frac{FP}{FP+TN}$$

where True Positives (TP) are the correctly predicted positive categories; False Positives (FP) are the incorrectly predicted positive categories; False Negatives (FN) are the incorrectly predicted negative categories; True Negatives (TN) are the correctly predicted negative categories.

## 3. Results and Analysis

### 3.1. Pre-Enhancement of Sound Samples

**Figure 7:**
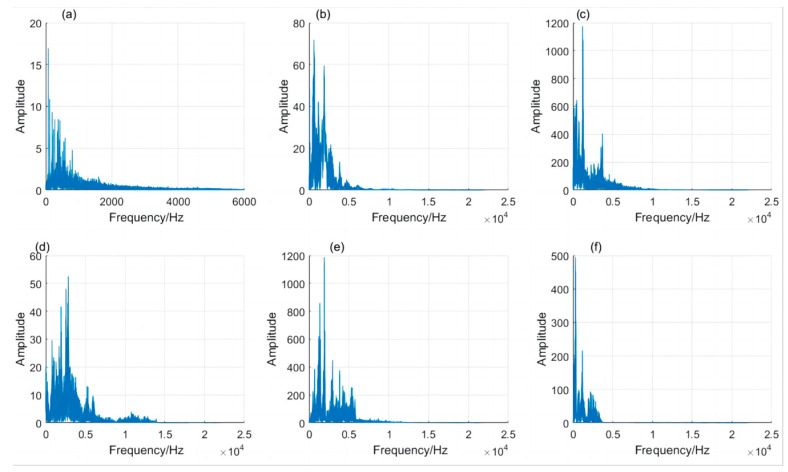
Frequency spectrum of pig farm noise and five kinds of pig sounds. (**a**) Spectrum of the noise signal, (**b**) Spectrum of eating sound signal, (**c**) Spectrum of estrous sound signal, (**d**) Spectrum of panting signal, (**e**) Spectrum of howling signal, (**f**) Spectrum of humming signal.

**Figure 8:**
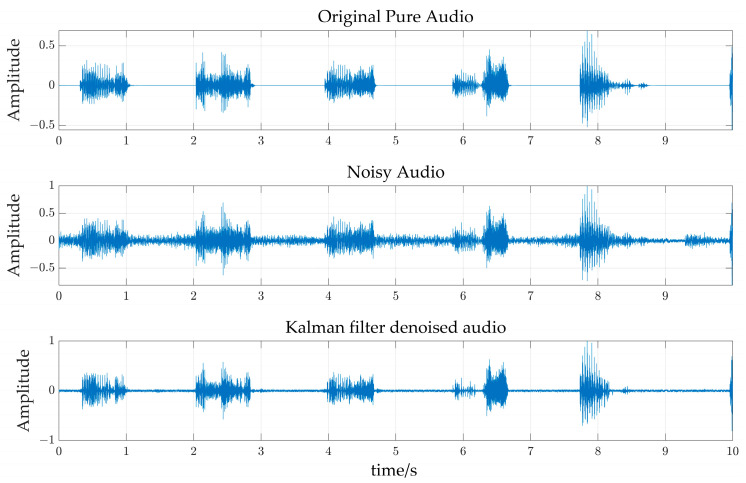
Comparison of waveforms before and after denoise.

In order to reduce the impact of noise signals such as exhaust fans, birds singing and the current sound of recording equipment on pig sound recognition, the data samples were filtered and denoised. Figure 7 shows the noise spectrum diagram of the pig farm environment and the sound signal spectrum diagram of five kinds of pig states in the experiment. It can be seen from the figure that the frequency band of the noise signal is mainly below 3 kHz, while the frequency band of pig sound signals (eating sounds, estrous sounds, panting, howling and humming) under the five states are 0–6, 0–9, 0–14, 0–6 and 0–4 kHz, respectively, which overlapped with the noise signal. So, the traditional digital filters (such as low-pass, high-pass or band-pass) have difficulty in removing the noise effectively. In order to quantitatively measure the denoising effect of the Kalman filter, this study selected a segment of relatively clean sound as the original sound. A section of noise was extracted and overlaid onto the original sound to create a noisy sound with a controllable signal-to-noise ratio (SNR). The noisy sound was subjected to denoising using the Kalman filter. By comparing the SNR of the sound before and after the denoising process, the denoising effect was quantitatively assessed. As shown in Figure 8, through Matlab simulation experiments, after adding pure sound signal to noise and setting the signal-to-noise ratio of the noisy sound to 5 dB, then denoising it with Kalman filtering, the denoised signal was compared with the original pure signal. The signal-to-noise ratio became 7.86 dB, indicating that the signal-to-noise ratio improved by 2.86 dB after denoising, effectively eliminating the interference of noise. However, as can be observed from Figure 8, it is also evident that while a significant amount of noise is removed, the original pig sound signal is also affected. This is inevitable in current-stage noise reduction techniques, as there are certain shared characteristics between noise and pig sounds. When removing the noise, segments of sound that share similarities with the noise’s characteristics are also eliminated. Such issues will receive greater attention in future research.

The improved EMD-TEO cepstral distance endpoint detection algorithm was used to intercept the appropriate non-silent segment sound, and the 39-d MFCC features were extracted to construct the sound recognition model.

### 3.2. Test Results and Comparative Analysis of Pig Sound Model

**Figure 9:**
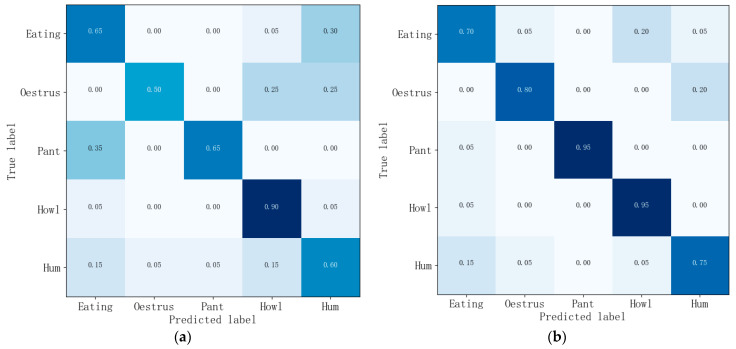
Confusion matrix for recognition of five kinds of pig sounds under two models. (**a**) Confusion matrix for GMM-HMM models, (**b**) Confusion matrix for DNN-HMM models. The darker the blue in the graph, the larger the value.

Figure 9 shows the confusion matrices for the five pig sounds under the GMM-HMM and DNN-HMM models. In order to examine the recognition ability of the DNN-HMM model, this study used Base-HMM as the baseline model and conducted ablation experiments. As shown in Table 1, the average recall, accuracy and specificity of Base-HMM for the five behaviors are 65.32%, 61% and 87.32%, respectively. The average recall, accuracy and specificity of GMM-HMM for the five behaviors are 70.94%, 66% and 91.66%, respectively. The average recall, accuracy and specificity of DNN-HMM for the five behaviors are 83.36%, 83% and 95.80%, respectively. Compared with Base-HMM, the average recall, accuracy, and specificity of DNN-HMM for the five behaviors improved by 18%, 22%, and 8.5%, respectively. Compared to GMM-HMM, DNN-HMM improved the average recall, accuracy and specificity of the five pig audios by 12.4%, 17% and 4.2%, respectively. The recall, accuracy and specificity of DNN-HMM are better than those of Base-HMM and GMM-HMM models, both individually and as a whole. Because DNN can learn deep nonlinear feature transformation, and the context information of the frame is introduced in the training of DNN in this study, the recognition is improved compared with GMM. According to the resulting indicators, DNN-HMM did not perform much better than GMM-HMM in the recognition of certain kinds of sound signals. The reason may be that the original GMM-HMM already had a high recognition rate for those low-complexity sound signals, leaving very little room for improvement that can be achieved by the relatively simple neural network combined with HMM. However, the differences in recall rate, accuracy, and specificity between DNN-HMM and GMM-HMM may be caused by the different complexities of the five types of pig sounds and the small number of training samples and features selected. So, the number of samples will be increased, and other characteristic parameters will be included as the input of the network to conduct further research on pig sound recognition.

In order to verify the robustness of the model, this study introduced AudioSet, a public dataset used for model performance validation, and compared it with the classical machine learning model SVM (support vector machine) [41] and the classical deep learning model ResNet18 [42], where the inputs were 39-dimensional MFCC feature vectors, and the hardware equipment used for training and each training parameter were kept the same. The AudioSet public dataset [43] was released by Google and contains a total of about 2.2 million 10 s long clips. In this study, a subset of the AudioSet dataset was used for the experiments, and the selected samples contained only one label and were 10 s long. Ten animal audio categories were selected for the study, namely horse, pig, cow, sheep, bird, cat, dog, wolf, tiger and frog, each category contained 200 samples and was randomly divided into a training set and a test set according to the ratio of 8:2, where the training set contained 1600 audio samples and the test set contained 400 samples, and the results are shown in Table 2.

As can be seen from Table 2, in the AudioSet subset of the public dataset used in the study, DNN-HMM has better evaluation metrics associated with it compared to the classical models SVM and ResNet18. The recall of DNN-HMM is 79.8%, which is 7.3% and 3.9% better than SVM and ResNet18, respectively. The accuracy of DNN-HMM is 79%, which is 8% and 4% better than SVM and ResNet18, respectively. The specificity of DNN-HMM is 94.5%, which is 1.3% and 0.7% higher than SVM and ResNet18, respectively. Since the AudioSet dataset was extracted from YouTube videos with varying types of background noise, it was not possible to perform effective noise reduction, which is one of the reasons for the low recognition accuracy.

**Figure 10:**
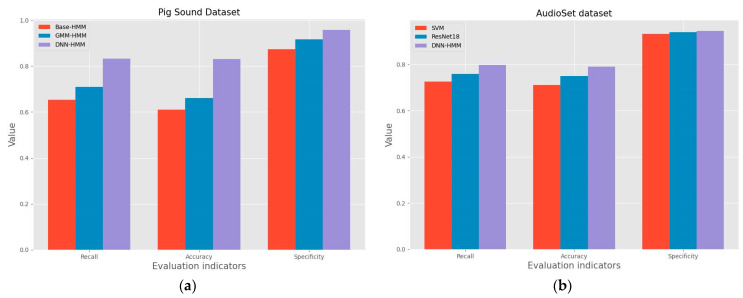
Data visualization of evaluation indicators. (**a**) Data visualization of ablation test evaluation indicators, (**b**) Data visualization of evaluation metrics for comparative testing of public datasets.

In order to make the data in Table 1 and Table 2 more intuitive, the corresponding test results are represented by bar charts. Figure 10a shows the ablation test results of the pig sound dataset, and Figure 10b shows the comparative test results of the public dataset AudioSet, which intuitively shows that the DNN-HMM model proposed in this study performs better.

## 4. Conclusions

This study proposes a new pig sound recognition method, which combines DNN instead of the Gaussian mixture matrix in the traditional sound recognition model with HMM. The collected pig sounds were denoised by a Kalman filter before extracting MFCCs, HMM was used to describe the dynamic changes in sound signals, and DNN was used to estimate the probability of observation features. The characteristic parameters corresponding to pig sounds were taken as the observation sequence. The phonemes of pig sounds were determined as the hidden states, and the pig sound recognition system of DNN-HMM was constructed. The experimental results demonstrate that the DNN-HMM model can effectively recognize a wide range of pig squeals compared with the baseline model Base-HMM and the traditional GMM-HMM model. It also outperforms the classical machine learning model SVM and the deep learning model ResNet18 on the public dataset AudioSet.

However, the sound samples used in the present study were all single-pig sounds, while in practice, the samples may be the mixed sounds of many pigs, which will make the sound feature extraction and recognition more difficult. In addition, we still heard some noises in sound signals after filtering, so how to effectively extract the sound features and filter out the noise is a problem that needs to be solved in the future.

As laboratory research on pig sound recognition is still in its infancy, the number of samples is small, there are not many sample categories, and there is a lack of previous experience. With the development of deep learning, our lab has carried out research on pig sound recognition, feature fusion and pig sound spectrogram recognition based on CNN and LSTM. However, the experiments have not been completed and will not be repeated here.

## Figures and Tables

**Table 1:** Results of ablation experiments modeled in the pig sound dataset.

Models	Recall	Accuracy	Specificity
Base-HMM	0.653	0.61	0.873
GMM-HMM	0.709	0.66	0.916
DNN-HMM	0.833	0.83	0.958

**Table 2:** Comparison of the performance of each model on the AudioSet dataset.

Models	Recall	Accuracy	Specificity
SVM	0.725	0.71	0.932
ResNet18	0.759	0.75	0.938
DNN-HMM	0.798	0.79	0.945

## Data Availability

Data are contained within the article.
